# Strategies to combat antimicrobial resistance: anti-plasmid and plasmid curing

**DOI:** 10.1093/femsre/fuy031

**Published:** 2018-07-30

**Authors:** Michelle M C Buckner, Maria Laura Ciusa, Laura J V Piddock

**Affiliations:** Institute of Microbiology and Infection, College of Medical and Dental Sciences, The University of Birmingham B15 2TT, UK

**Keywords:** antimicrobial resistance, plasmid, plasmid curing, CRISPR/Cas, antibiotics, conjugation inhibitors

## Abstract

Antimicrobial resistance (AMR) is a global problem hindering treatment of bacterial infections, rendering many aspects of modern medicine less effective. AMR genes (ARGs) are frequently located on plasmids, which are self-replicating elements of DNA. They are often transmissible between bacteria, and some have spread globally. Novel strategies to combat AMR are needed, and plasmid curing and anti-plasmid approaches could reduce ARG prevalence, and sensitise bacteria to antibiotics. We discuss the use of curing agents as laboratory tools including chemicals (e.g. detergents and intercalating agents), drugs used in medicine including ascorbic acid, psychotropic drugs (e.g. chlorpromazine), antibiotics (e.g. aminocoumarins, quinolones and rifampicin) and plant-derived compounds. Novel strategies are examined; these include conjugation inhibitors (e.g. TraE inhibitors, linoleic, oleic, 2-hexadecynoic and tanzawaic acids), systems designed around plasmid incompatibility, phages and CRISPR/Cas-based approaches. Currently, there is a general lack of *in vivo* curing options. This review highlights this important shortfall, which if filled could provide a promising mechanism to reduce ARG prevalence in humans and animals. Plasmid curing mechanisms which are not suitable for *in vivo* use could still prove important for reducing the global burden of AMR, as high levels of ARGs exist in the environment.

## Teaser

Removing plasmids from bacteria in different ecosystems could be an important aspect of fighting antimicrobial resistance.

## INTRODUCTION

One of the major threats facing society is the rise in number of antimicrobial-resistant (AMR) bacteria (O’Neill 2016). Antimicrobials underpin modern medicine; they are used to treat infections, to prevent infections (prophylaxis) during medical procedures (e.g. surgery) and they are crucial for patients with compromised immune function (Holmes *et al.*^2016^; Laxminarayan *et al.*^2016^). Between 2000 and 2010, global human use of antibiotics increased by 36%, and the use of two last-resort antibiotics, carbapenems and polymyxins, increased by 45% and 13%, respectively (Van Boeckel *et al.*^2014^). Antimicrobials have many non-human uses including in animals for growth promotion, veterinary treatment and aquaculture (Cabello 2006; Meek, Vyas and Piddock 2015; Van Boeckel *et al.*^2015^). In 2013, an estimated 131 109 tons of antimicrobials were used globally in food animals; by 2030 this is expected to increase to 200 235 tons (Van Boeckel *et al.*^2017^). However, there is a growing trend to improve antimicrobial stewardship in many countries. For example, in Switzerland veterinary antimicrobial sales increased between 2006 and 2008, but then steadily decreased, reaching a 26.2% reduction in 2013 (Carmo *et al.*^2017^). In addition to human and animal use, many cleaning and personal hygiene products contain biocides, such as triclosan, which can select for mutants resistant to biocides, and in some cases to antibiotics used in medicine (Meek, Vyas and Piddock 2015; Webber *et al.*^2015^, ^2017^).

A key factor that has led to the rise and global dissemination of multidrug-resistant (MDR) bacteria are mobile antimicrobial resistance genes (ARGs). These are frequently located on plasmids, which are pieces of usually circular, self-replicating DNA which can code for a variety of different functional gene groups. Aspects of plasmid biology have been extensively reviewed elsewhere, but, in brief, plasmids often include partitioning systems, toxin–antitoxin (TA) systems and conjugative/transmission systems (Van Melderen and Saavedra De Bast 2009; Pinto, Pappas and Winans 2012; Carattoli 2013; Baxter and Funnell 2014; Goessweiner-Mohr *et al.*^2014^; Kado 2014; MacLean and San Millan 2015; Ruiz-Maso *et al.*^2015^; Cabezon *et al.*^2015^; Ilangovan, Connery and Waksman 2015; Chan, Espinosa and Yeo 2016; Banuelos-Vazquez, Torres Tejerizo and Brom 2017; Hall *et al.*^2017^; Hulter *et al.*^2017^). Conjugation is mediated by type IV secretion coupled with a relaxosome complex to mediate DNA movement from one cell to another (Ilangovan, Connery and Waksman 2015).

Plasmids are frequently categorised based on incompatibility groups (Inc), defined as the inability of two related plasmids to be propagated stably in the same cell and may be due to competition for the same replication or segregation sites, or caused by repression of replication initiation (Novick 1987; Carattoli 2009). Reviews on incompatibility groups and plasmid classification can be found elsewhere (Novick 1987; Carattoli 2011; Shintani, Sanchez and Kimbara 2015; Orlek *et al.*^2017^). Plasmids that share the same mechanisms for replication or partitioning are placed in the same incompatibility groups. Plasmid incompatibility has been used to follow the movement and evolution of plasmids conferring AMR (Carattoli *et al.*^2005^).

**Figure 1.**
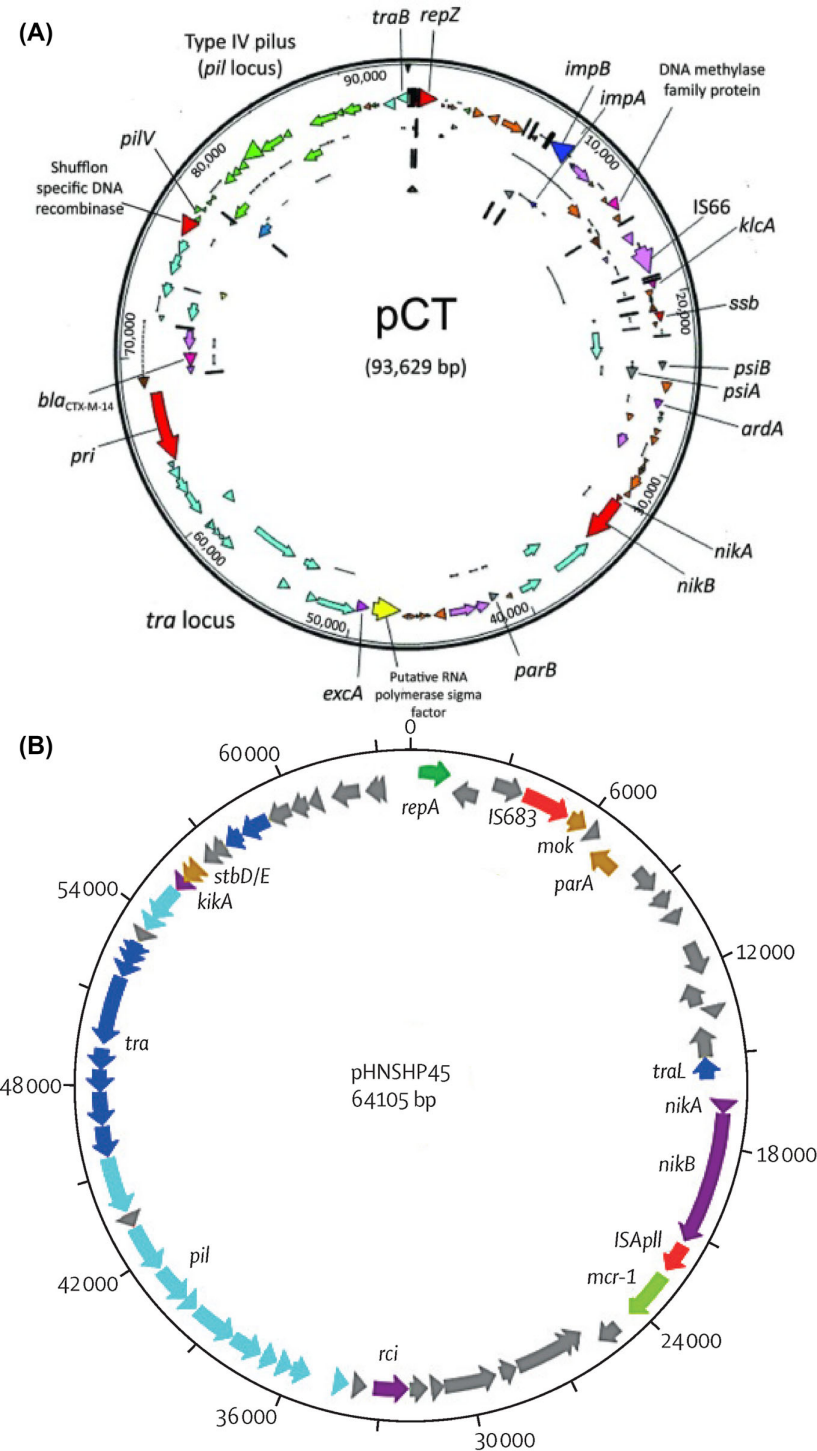
Organisation of two antibiotic resistance plasmids. (**A**) pCT_CTX-M_ (IncK). Brown, pseudogenes; orange, hypothetic proteins; light pink, insertion sequences; light blue, *tra* locus; green, *pil* locus; dark pink, antimicrobial drug resistance gene; yellow, putative sigma factor; red, replication-associated genes. Arrows show the direction of transcription. Reproduced with permission from Cottell *et al.* (2011). (**B**) pHNSHP45_mcr-1_. Light blue, type IV pilus; dark blue, transfer region; yellow, plasmid stability; dark green, plasmid replication; red, insertion sequence; light green, antimicrobial resistance; purple, other proteins; grey, hypothetical proteins. Reproduced with permission from Liu *et al.* (2016).

ARGs that pose a serious threat to human medicine are typically found in Gram-negative bacteria. These include genes coding for extended spectrum β-lactamases (ESBL) (e.g. CTX-M), carbapenemases (e.g. KPC, NDM and OXA-58) (Holmes *et al.*^2016^) and colistin resistance (e.g. MCR-1) (Liu *et al.*^2016^). The issues surrounding AMR plasmids are derived in part by their substantial complexity. Plasmids often display a high degree of plasticity, with frequent insertions, deletions and rearrangements of DNA including changes to specific ARGs (Kado 2014). For example, the *bla*_CTX-M_ gene is highly variable, and the CTX-M family of ESBLs are commonly coded for by multiple different plasmids, such as pCT (Fig. 1A) (Cottell *et al.*^2011^, Bevan, Jones and Hawkey 2017). According to the Beta-Lactamase DataBase, 207 variants of *bla*_CTX-M_ have been identified (accessed on 11 May 2018) (Naas *et al.*^2017^). Another example of a plasmid-mediated ARG is the *mcr-1* gene, first identified on a transmissible plasmid, pHNSHP45, in 2016 (Fig. 1b) (Liu *et al.*^2016^). Since then *mcr-1* and variants of this gene have been identified on multiple plasmid backbones and host strains. Of concern are isolates carrying colistin and carbapenem ARGs, as few treatment options would remain for infections caused by such bacteria (Lai *et al.*^2017^; Wang *et al.*^2017^; Zhou *et al.*^2017^). In addition to these examples, plasmids can carry a variety of other resistance genes, including *qnr* variants, *aac*(6΄)*-lb-cr* and plasmid-mediated efflux pump genes such as *oqxAB* and *qepA*, which confer low levels of resistance to quinolone antimicrobials (Jacoby, Strahilevitz and Hooper 2014). Increasingly, research should focus on ARGs which are frequently mobilised and transmit between bacteria (Crofts, Gasparrini and Dantas 2017).

In the European Union, resistance to carbapenem antibiotics in invasive *K*lebsiella *pneumoniae* isolates ranges from 66.9% (Greece), 33.9% (Italy), 2.1% (Spain) to <5% (Northern Europe) (ECDC 2016). For invasive *E. coli* infections, resistance to third-generation cephalosporins ranges from 5% in Iceland to 50% in Italy, Slovakia and Bulgaria, while carbapenem resistance in *E. coli* is <1% for most of the EU and between 1–5% for Romania (ECDC 2016). A study of travellers returning to the Netherlands found 30.5% of participants had ESBLs in their bacterial flora, while only 8.6% had ESBLs before their trip (Paltansing *et al.*^2013^). A large prospective study of 2001 Dutch travellers found 34.7% with no ESBL producing *Enterobacteriaceae* prior to international travel returned with ESBL producing strains (Arcilla *et al.*^2017^). A similar study of 188 Swedish travellers found 32% returned from regions associated with high levels of ESBL producing *Enterobacteriaceae* carrying these antibiotic-resistant bacteria (Vading *et al.*^2016^). One isolate contained both *bla*_CTX-M_ and *mcr-1* (Vading *et al.*^2016^). Indeed, *mcr-1* was detected by metagenomics in 4.9% of faecal samples from 122 healthy Dutch travellers upon return from travel to South/East Asia and/or Southern Africa undertaken between 2011 and 2012 (von Wintersdorff *et al.*^2016^). However, in this study little is known about the index isolate in which the *mcr-1* gene originated, including the isolate's susceptibility profiles. Therefore, it is possible that the isolates were susceptible to other antimicrobials. In the majority of studies travellers who obtained ESBL-producing bacteria eventually lost the ESBL genes upon return. Of 15 Swiss volunteers, 3 were colonised by ESBL-resistant *Enterobacteriaceae* before their trip, all were colonised upon return and 6 were still colonised 6 months post-travel (Pires *et al.*^2016^). Of the resistant isolates 80% contained IncF family plasmids, and in some of the participants who were colonised 6 months after travel, the plasmids had moved into new host bacteria (Pires *et al.*^2016^). *bla*_CTX-M-15_ was the most prevalent ESBL, comprising 92% of the ESBL producers immediately after travel (Pires *et al.*^2016^). Together, this highlights the need to reduce the prevalence of ARGs on a global scale.

### Could plasmid curing be a strategy to reduce AMR?

Plasmid curing is the process by which plasmids are removed from bacterial populations. This is an attractive strategy to combat AMR as it has the potential to remove ARGs from a population while leaving the bacterial community intact. This means, for example, that the structure of the gastrointestinal microbiome of a chicken treated with a plasmid curing agent might remain largely unchanged, but potentially pathogenic bacteria which may unfortunately be transmitted into the food chain would be susceptible to antibiotics. Alternatively, a plasmid curing agent could be given to a patient prior to surgery, to reduce the likelihood of a resistant hospital acquired infection. Plasmid curing agents could also be taken by international travellers to reduce the global spread of AMR. Unfortunately, at the moment no such treatment options are in use. In fact, there are very few curing mechanisms that have been tested *in vivo*, even in experimental models. Therefore, research in this area is urgently needed. Recently, it was shown that 24% of non-antibacterial drugs impact growth of members of the human microbiome (Maier *et al.*^2018^). Studies such as this would be important for determining any impact of anti-plasmid compounds on the microbiome.

The ‘One Health’ approach to tacking AMR is based around the notion that AMR does not abide by human, animal or political boundaries, and therefore a multisectoral and multifaceted approach is required. Likewise, anti-plasmid strategies should also adopt a One Health strategy, and not be focused on human medicine alone. Indeed some anti-plasmid strategies are unsuitable or unviable for human use. Furthermore, anti-plasmid strategies alone will never ‘solve’ AMR; nonetheless, they could play an important role in reducing global resistance levels. Removing drug-resistance plasmids is a strategy for all sectors to reduce the overall burden of AMR. For example, plasmid curing could be used to remove ARGs from bacteria in sewage before release into the environment. Human and animal waste is often recycled and used to fertilise agricultural land; this can contain high concentrations and varieties of ARGs which can be passed on to people (Meek, Vyas and Piddock 2015; Rahube *et al.*^2016^). One study performed in Canada found in the first year vegetables grown above, on and below the surface of soil treated with sewage contained significantly more ARGs than non-treated soil (Rahube *et al.*^2016^). An abundance of ARGs were detected in plasmid metagenome libraries constructed from the influent, activated sludge and digested sludge from two wastewater treatment plants in Hong Kong, demonstrating that these were important reservoirs of ARGs (Li, Li and Zhang 2015). River samples taken upstream and downstream of a tertiary waste water treatment plant in the UK in 2009 and 2011 were examined for third-generation cephalosporin-resistant *Enterobacteriaceae* (Amos *et al.*^2014^). Significantly higher amounts of *bla*_CTX-M-15_ were found downstream of the plant, and 10 novel genetic contexts were identified (Amos *et al.*^2014^). The plasmids containing *bla*_CTX-M-15_ were conjugative, and were in pathogens such as the highly successful extraintestinal *E. coli* ST131 (Stoesser *et al.*^2016^), and other species never before reported to carry *bla*_CTX-M-15_ (Amos *et al.*^2014^). IncP-1ε plasmids were detected in manure and arable soil in Germany, and a correlation was found between the presence of IncP-1ε plasmids and antibiotic use (Heuer *et al.*^2012^). A waste water treatment plant in Brazil found 34% of *E. coli* and 27% of *K. pneumoniae* were resistant to cephalosporins and/or quinolones, and 5.4% of *Klebsiella* species were carbapenem resistant in raw as well as treated water (Conte *et al.*^2016^). Analysis of these ARGs showed a high prevalence of *bla*_CTX-M_ and *bla*_SHV_ (Conte *et al.*^2016^). Recent work from our group examined wastewater used for irrigation of urban agriculture plots in Burkina Faso. This wastewater contained multiple ARGs including ESBLs, 10 different *Enterobacteriaceae*-associated plasmid incompatibility groups and 30 Gram-positive replicons associated with ARGs (Bougnom *et al.*, submitted). Together, these studies demonstrate that a treatment such as plasmid curing agents to remove ARGs from manure, sewage and waste water are needed.

The search for plasmid curing compounds began decades ago, and gained momentum in the 1970s (Table 1). The number of publications peaked in the 1980s (based on searches for publications relating to plasmid curing performed on NCBI PubMed). However, most compounds were toxic, and would produce adverse or unwanted side effects and thus had little use in human medicine. This was followed by a decline in interest and publications. Generally, plasmid curing properties have been evaluated by culturing strains in the presence of a compound or extract at subgrowth inhibitory concentrations. Curing effects are then confirmed by the reversal of plasmid-mediated antibiotic resistance and/or by physical loss of the plasmid(s). Therefore, many of the older publications only refer to the loss of an AMR phenotype.

**Table 1.** Plasmid curing compounds.

Curing Agent	Species	Plasmid Cured	Key Findings	Reference
Acridine orange	*E. coli*	Small plasmids (UTI isolates)	75 μg/mL: 11.76% CF for plasmids ≤2.7 mDa	Zaman, Pasha and Akhter (2010)
		pBR322	100 μg/mL: 35% CF	Keyhani *et al.* (2006)
		pBR325	100 μg/mL: 15% CF	Keyhani *et al.* (2006)
		pUK657	375 μg/mL: 14.28% CF	Beg and Ahmad (2000)
	*V. parahaemolyticus*	AMR plasmid	0.2 mg/mL cured 6/13 plasmids from isolates (1.2–10kb).	Letchumanan *et al.* (2015)
	*L. plantarum*	Raffinose & lactose metabolising plasmid	0.1 mg/mL cured 10/12 plasmids	Adeyemo and Onilude (2015)
	*S. aureus*	Staphyloccocin plasmid	15 μg/mL: 12.1% CF	Jetten and Vogels (1973)
		pED503	15 μg/mL: 3.4% CF	Ersfeld-Dressen, Sahl and Brandis (1984)
	*B. fragilis*	AMR plasmid	16 μg/mL cured resistance to Ery and Clin	Rotimi, Duerden and Hafiz (1981)
	*B. thetaiotaomicron*	AMR plasmid	16 μg/mL cured resistance to Ery and Clin	Rotimi, Duerden and Hafiz (1981)
Acriflavine	*S. enterica*	AMR plasmids	Of plasmids with five resistance phenotypes, 35% CF of *S.* Oranienburg, 5% CF of *S.* Panama. Of plasmids with one resistance phenotype, 98% CF of *S.* Panama and *S.* paratyphi B	Bouanchaud and Chabbert (1971)
	*E. coli*	AMR plasmid	Three plasmids cured at 5, 12 and 22% CF	Bouanchaud and Chabbert (1971)
		Haemolysin producing plasmids	24 h incubation with 10 μg/mL resulted in low CF	Mitchell and Kenworthy (1977)
	Group A *Streptococci*	AMR plasmid	0.2 μg/mL for 18 h: 2.1%–4.3% CF of three plasmids	Nakae, Inoue and Mitsuhashi (1975)
	*L. casei*	pDR101	10 μg/mL for 48 h: 7.2% CF	Chassy, Gibson and Guiffrida (1978)
	*L. reuteri*	pLUL631 (lactose fermenting)	2 μg/mL: 1%–10% CF	Axelsson *et al.* (1988)
	*S. aureus*	Staphyloccocin plasmids	2 μg/mL: 25% CF	Jetten and Vogels (1973)
	*B. fragilis*	AMR plasmid	16 μg/mL, 18–21 days: loss of Ery, Clin and Tet resistance plasmid	Rotimi, Duerden and Hafiz (1981)
	*B. thetaiotaomicron*	AMR plasmid	16 μg/mL, 18–21 days: loss of Ery, Clin and Tet resistance plasmid	Rotimi, Duerden and Hafiz (1981)
	*O. oeni*	pRS1, pRS2, pRS3	2.5–10 μg/Ml, CF of: 18.7% (pRS1), 6.2% (pRS2), 62.5% (pRS3), 31.2% (pRS2 & pRS3 simultaneously)	Mesas, Rodriguez and Alegre (2004)
	*E. faecium*	AMR plasmids	Sub-MIC levels resulted in cured isolates	Coleri *et al.* (2004)
	*E. faecalis*	AMR plasmids	Sub-MIC levels resulted in cured isolates	Coleri *et al.* (2004)
Ascorbic Acid	*S. aureus*	Penicillinase plasmid	1 mM for 6 h: 12%–35% CF	Amábile Cuevas (1988)
		Aminoglycoside resistance plasmid	1 mM for 6 h: 4 of six strains cured, with 10%–48% CF	Amábile-Cuevas, Piña-Zentella and Wah-Laborde (1991)
		pI55cI	1 mM for 6 h: 48% CF	Amábile-Cuevas, Piña-Zentella and Wah-Laborde (1991)
	*P. acidilactici*	Pediocin producing plasmid	1 mM: 35% CF of 7.8 kb plasmid	Ramesh, Halami and Chandrashekar (2000)
Bile	*S. enterica* Typhimurium	pSLT	15% ox bile: 10^−6^ frequency of plasmid loss in wild type. In *ccdB* mutant frequency was 10^−4^	García-Quintanilla *et al.* (2006)
	*S. enterica* Infantis	pESI	1%–4% bile: reduced CF	Aviv, Rahav and Gal-mor (2016)
Chlorpromazine	*E. coli*	F’lac plasmid	20–60 μg/mL: 5%–20% CF, most efficient at pH 7.6	Mandi *et al.* (1975)
		R-factor	50 μg/mL: plasmid curing was observed	Molnar, Mandi and Kiraly (1976)
		R114 plasmid	Enhanced curing activity with methylene blue	Molnar *et al.* (1980)
	*S. aureus*	QacA encoding plasmid	Successive passaging in 2–20 mg/mL resulted in curing	Costa *et al.* (2010)
Ethidium bromide	*S. aureus*	Penicillinase carrying plasmids	8 × 10^−6^M at pH 7.2: CF of 50% (maximum). 6 × 10^−6^M: CF average of 20%, ranging from 0.21%–58% depending on plasmid/strain. Curing peaked at 10–12 h, became refractory to additional curing	Bouanchaud, Scavizzi and Chabbert (1969); Rubin and Rosenblum (1971)
		Staphyloccocin producing plasmid	1.25 μg/mL: 94% CF	Jetten and Vogels (1973)
		pED503	3.6 μg/mL: 4.4% CF	Ersfeld-Dressen, Sahl and Brandis (1984)
		AMR plasmids	32% and 60% CF for Pen and mercury resistance plasmids	Bouanchaud and Chabbert (1971)
	*E. aerogenes*	pKpQIL-like (*bla*_TEM-1_ and *bla*_KPC-3_)	400–600 μg/mL: 85% CF	Pulcrano *et al.* (2016)
	*Salmonella*	AMR plasmids	100–2000 μg/mL for 1–7 days cured 2/17 strains	Poppe and Gyles (1988)
	*E. coli*	F’-lac plasmids	6–250 × 10^−5^M: 20% CF	Bouanchaud, Scavizzi and Chabbert (1969)
		p424	0.52 mM cured plasmid, four cured variants had altered colony morphology and biochemical modifications	Rosas *et al.* (1983)
		pUK651	200 μg/mL: 36.6% CF	Beg and Ahmad (2000)
		Haemolysin producing plasmids	50 μg/mL: low frequency of plasmid loss at 24 h	Mitchell and Kenworthy (1977)
		AMR plasmids	7.5 × 10^−5^ and 1.3 × 10^−3^M: CF of 71% and 32%, respectively	Bouanchaud, Scavizzi and Chabbert (1969)
		AMR plasmids	32% curing of resistance to five antibiotics	Bouanchaud and Chabbert (1971)
		UTI plasmids	125 μg/mL: 17.65% CF	Zaman, Pasha and Akhter (2010)
	*B. cereus*	Hydrocarbon degrading plasmid	100 μg/mL: cured isolates enabling testing of plasmid properties	Borah and Yadav (2015)
	*B. fragilis*	AMR plasmid	16 μg/mL cured Ery and Clin resistance. Curing of Tet resistance required 18–21 days	Rotimi, Duerden and Hafiz (1981)
	*B. thetaiotaomicron*	AMR plasmid	16 μg/mL cured Ery and Clin resistance. Curing of Tet resistance required 18–21 days	Rotimi, Duerden and Hafiz (1981)
Irgasan (Triclosan)	*E. coli*	pMIB4	100× below MIC cured plasmid. Effective in broth and embedded in silicone hydrogels	Riber *et al.* (2016)
Lawsone	*S. aureus*	Van resistance plasmid	200 μg/mL: 20% CF (1/2 MIC)	Jahagirdar, Patwardhan and Dhakephalkar (2008)
Plumbagin	*E. coli*	R6K	200 μg/mL: 42% CF of 2/6 resistance markers	Lakhmi, Padma and Polasa (1987)
		TP181	100 μg/mL: 100% CF	Lakhmi, Padma and Polasa (1987)
		R162	100 μg/mL: 100% CF	Lakhmi, Padma and Polasa (1987)
		TP154	100 μg/mL: 45% CF of 3/6 resistance markers	Lakhmi, Padma and Polasa (1987)
		RP4	12.5 μg/mL: 32% CF	Bharathi and Polasa (1991)
		pKT231	12.5 μg/mL: 10% CF	Bharathi and Polasa (1991)
		pTP181-derivatives	25 μg/mL: 11%–47% CFCaused by interference with plasmid replication and maintenance	Lakshmi and Thomas (1996)
		pUK651	7000 μg/mL: 14% CF (sub-MIC)	(Beg and Ahmad (2000)
		R plasmid	1000 μg/mL: 15% CF.	Patwardhan *et al.* (2015)
	*S. aureus*	Van resistance plasmid	25 μg/mL: 4% CF, 50 μg/mL inhibited growth	Jahagirdar, Patwardhan and Dhakephalkar (2008)
	*P. aeruginosa*	R plasmid	1000 μg/mL: 13% CF	Patwardhan *et al.* (2015)
	*P. vulgaris*	R plasmid	500 μg/mL: 32% CF	Patwardhan *et al.* (2015)
	*K. pneumoniae*	R plasmid	500 μg/mL: 30% CF	Patwardhan *et al.* (2015)
Promethazine	*E. coli*	AMR plasmid	Plasmids eliminated	Spengler *et al.* (2003)
		F’lac plasmid	At 37°C, 80 μg/mL: 79.6% CF At 39°C, 80 μg/mL: 88% CF Multi-species co-cultures reduced promethazine concentration required for curing	Molnár, Amaral and Molnár (2003); Spengler *et al.* (2003)
		pBR322	TF-14 (a potential proton pump inhibitor) increased promethazine CF	Wolfart *et al.* (2006)
Rifampicin	*E. coli*	Haemolysin plasmids	2 μg/mL, 24 h incubation led to high CF	Mitchell and Kenworthy (1977)
		F’lac	3–7.5 μg/mL resulted in curing. Rif/RNA polymerase interaction required for curing	Bazzicalupo and Tocchini-Valentini (1972)
	*S. aureus*	Penicillinase plasmid	0.1 μg/mL: 20% CF, 0.05 μg/mL: 5% CF	Johnston and Richmond (1970); Wood, Carter and Best (1977)
Sodium dodecyl sulphate (SDS)	*E. coli*	R and F factors	24 h of 10% SDS: 5.3%–22% CF, 72 h resulted in 95%–100% CF	Tomoeda *et al.* (1968)
		p424	10% cured variants had altered colony morphology and biochemical modifications	Rosas *et al.* (1983)
		pR4	100 μg/mL: 12.5% CF	Bharathi and Polasa (1991)
		pKT231	200 μg/mL: 7.5% CF	Bharathi and Polasa (1991)
		pBR322	0.25%–1%: 27%–35% CF	Keyhani *et al.* (2006)
		UTI plasmids	10% w/v: 7.4% CF	Zaman, Pasha and Akhter (2010)
	*K. pneumoniae*	Large indigenous plasmid (96 kb)	4% resulted in 1/8 colonies successfully cured	El-Mansi *et al.* (2000)
	*Lactobacillus* isolates (milk)	AMR plasmids	1% cured 5 of 7 isolates	Lavanya *et al.* (2011)
	*P. aeruginosa*	pBC15	10% was effective	Raja and Selvam (2009)
	*S. aureus*	Staphyloccocin producing plasmid	30 μg/mL: 100% CF	Jetten and Vogels (1973)
Thioridazine	*E. coli*	AMR plasmid	75% MIC eliminated resistance	Radhakrishnan *et al.* (1999)
	*S. flexneri*	AMR plasmid	75% MIC eliminated resistance	Radhakrishnan *et al.* (1999)
	*V. cholera*	AMR plasmid	75% MIC eliminated resistance	Radhakrishnan *et al.* (1999)
Trifluoperazine	*E. coli*	AMR plasmid	Reviewed in detail by	Spengler *et al.* (2003)
1΄-acetoxychavicol acetate	*E. coli*	pAR1813	400 μg/mL: 32% CF	Latha *et al.* (2009)
		RP4	400 μg/mL: 7% CF	Latha *et al.* (2009)
	*S.* Typhi	pAR1814	800 μg/mL: 75% CF	Latha *et al.* (2009)
	*P. aeruginosa*	pAR1816	800 μg/mL: 75% CF	Latha *et al.* (2009)
	*E. faecalis*	pAR1812	400 μg/mL: 66% CF	Latha *et al.* (2009)
	*B. cereus*	pAR1817	400 μg/mL: 6% CF	Latha *et al.* (2009)
8-epidiosbulbin E acetate	*E. coli*	RP4	25 μg/mL: 44% CF	Shriram *et al.* (2008)
		pARI813	25 μg/mL: 44% CF	Shriram *et al.* (2008)
	*B. subtilis*	pUB110	100 μg/mL: 48% CF	Shriram *et al.* (2008)
	*P. aeruginosa*	RMS163	200 μg/mL: 30% CF	Shriram *et al.* (2008)
		RIP64	100 μg/mL: 64% CF	Shriram *et al.* (2008)
	*E. faecalis*	pARI812	200 μg/mL: 48% CF	Shriram *et al.* (2008)
	*S. sonnei*	pARI815	25 μg/mL: 32% CF	Shriram *et al.* (2008)

CF—Curing Frequency: the proportion of colonies which were cured of the plasmid compared to non-cured colonies. Ery—erythromycin, Clin—clindamycin, Tet—tetracycline, Pen—penicillin, Van—vancomycin, Rif—rifampicin.

The rise in AMR, specifically plasmid-mediated resistance, combined with the dwindling pipeline of new drugs in development has resulted in a resurgence of interest in plasmid curing. Strategies of plasmid curing vary greatly, such as the use of chemicals, drugs, natural products, phage therapies, other plasmids and even CRISPR/Cas. A recent study demonstrated that inhibiting plasmid conjugation was an effective means to remove a plasmid from a bacterial population over time (Lopatkin *et al.*^2017^). The authors concluded that strategies to prevent plasmid conjugation should be explored as a means to reduce AMR plasmid prevalence (Lopatkin *et al.*^2017^). Plasmid curing of a population can also occur when plasmid replication is prevented or reduced, or if plasmid segregation is disrupted, resulting in gradual reduction in plasmid carrying cells. Plasmid curing can also be achieved by increasing the fitness cost associated with plasmid carriage. We anticipate over the next decade that these mechanisms will be studied, streamlined and new practical ways to reduce global AMR plasmid carriage, and hence presence of ARGs, will be developed.

## PLASMID CURING COMPOUNDS

Many compounds have shown some plasmid curing activity. These include detergents, biocides, DNA intercalating agents, antibiotics (e.g. aminocoumarins, quinolones, rifampicin), ascorbic acid, psychotropic drugs (e.g. chlorpromazine) and plant-derived compounds (Table 1). The effectiveness of these compounds varies greatly and depends on bacterial strain, plasmid and growth conditions. Plasmid curing compounds can act through different mechanisms. In many cases, the compound disrupts plasmid replication by integrating into the DNA (e.g. intercalating agents and chlorpromazine), causing breaks in DNA (e.g. ascorbic acid) or by influencing plasmid supercoiling (e.g. aminocoumarins and quinolones). Plasmid curing compounds can also act by preventing conjugation (e.g. unsaturated fatty acids and TraE inhibitors). Each of these can result in reduced plasmid prevalence within the population over time. The mechanism of action of some curing agents remains to be fully elucidated. One could hypothesise that plasmid curing compounds could also target plasmid segregation, by preventing equal distribution among daughter cells, or increase the fitness burden associated with plasmid carriage.

### Detergents

The detergents bile and sodium dodecyl sulphate (SDS) are able to cure some plasmids from some bacterial strains (Table 1). Four notable examples include a study where bile salts dose-dependently caused the loss of the *Salmonella enterica* serovar Typhimurium virulence plasmid, pSLT (García-Quintanilla *et al.*^2006^). However, the level of bile required was 10%–15%, which is significantly higher than that found normally within the small intestine (0.2%–2%) (García-Quintanilla *et al.*^2006^; Kristoffersen *et al.*^2007^). The *Salmonella* virulence plasmid can be transmitted to new hosts in the mouse intestine, but transmission is unlikely to occur in areas with high levels of bile (García-Quintanilla, Ramos-Morales and Casadesús 2008). Bile (>1%) reduced expression of conjugative pilus genes *pilV* and *pilT*, and decreased conjugation of *S. enterica* Infantis mega plasmid pESI (280 kb), encoding resistance to tetracycline, sulfamethoxazole and trimethoprim as well as virulence traits (Aviv *et al.*^2014^; Aviv, Rahav and Gal-mor 2016). The relevance of bile-mediated plasmid curing during human *Salmonella* infections remains unclear. In addition, the levels of bile required for plasmid curing or to reduce plasmid transmission may result in diarrhoea, and therefore bile is unlikely to be used as a treatment.

SDS-based plasmid curing methods have been used as a laboratory tool for decades. In 1968, SDS was shown to reduce carriage of fertility and resistance factors (F and R factors/plasmids) (Tomoeda *et al.*^1968^). Over the years, SDS has been used to cure plasmids from *E. coli* (Rosas *et al.*^1983^; Bharathi and Polasa 1991; Keyhani *et al.*^2006^; Zaman, Pasha and Akhter 2010), *K. pneumoniae* (El-Mansi *et al.*^2000^), *Pseudomonas aeruginosa* (Raja and Selvam 2009), *Lactobacillus* species (Lavanya *et al.*^2011^) and *Staphylococcus aureus* (Jetten and Vogels 1973) (Table 1). SDS also had other effects on bacteria; these included changes in the peptidoglycan layer, bacterial cell size, septation and loss of outer membrane components (Rosas *et al.*^1983^).

In summary, detergents are unlikely to be used in humans or animals to reduce AMR plasmids, mainly due to the high concentrations needed, and the associated unwanted gastrointestinal side effects, such as SDS-induced colitis. However, detergents continue to be used in the laboratory setting as a tool to study plasmid biology.

### Biocides

Recently, it was shown that concentrations well below the MIC of triclosan (also called irgasan) increased the loss of a GFP reporter plasmid pMIB4 from *E. coli* (Riber *et al.*^2016^). A key finding of this paper was that triclosan embedded in interpenetrating polymer networks of silicone hydrogels was effective at reducing plasmid carriage. The use of such technology as a drug delivery system is appealing, especially for items such as indwelling medical devices (e.g. catheters). However, exposure to triclosan can select for MDR bacterial mutants, largely due to overexpression of bacterial efflux pumps (Chuanchuen *et al.*^2001^; Webber et al., 2008, 2015; Hernandez *et al.*^2011^; Fernando *et al.*^2014^; Rensch *et al.*^2014^; Gantzhorn, Olsen and Thomsen 2015). Therefore, caution should be used implementing such a strategy.

### DNA intercalating agents

The DNA intercalating agents acridine orange, ethidium bromide and acriflavine also have plasmid curing properties. Acridine orange cured *E. coli* (Keyhani *et al.*^2006^; Zaman, Pasha and Akhter 2010), *Vibrio parahaemolyticus* (Letchumanan *et al.*^2015^), *Lactobacillus plantarum* (Adeyemo and Onilude 2015), *S. aureus* (Jetten and Vogels 1973; Ersfeld-Dressen, Sahl and Brandis 1984)*, Bacteroides fragilis* and *B. thetaiotaomicron* (Rotimi, Duerden and Hafiz 1981) (Table 1). In the late 1960s and early 1970s, ethidium bromide was found to eliminate plasmids from various strains of *S. aureus* and *Escherichia coli* (Table 1) (Bouanchaud, Scavizzi and Chabbert 1969; Rubin and Rosenblum 1971). More recently, it has been used to cure other plasmids from *E. coli* (Rosas *et al.*^1983^), *Bacillus cereus* (Borah and RNS 2015), clinical *Enterobacter aerogenes* isolates of a *bla*_TEM-1_ and *bla*_KPC-3_ pKpQIL-like plasmid (Pulcrano *et al.*^2016^), and cured plasmids from two avian *Salmonella* strains (Poppe and Gyles 1988) (Table 1).

Acriflavine cured some resistance plasmids from *Salmonella* Oranienburg, *S.* Panama and *E. coli* K12 *in vitro* and in a murine *in vivo* model (Bouanchaud and Chabbert 1971). Acriflavine also cured *E. coli* of haemolysin production (Mitchell and Kenworthy 1977). However, it is more commonly associated with curing of Gram-positive bacteria. It cured plasmids from Group A *Streptococci* (Nakae, Inoue and Mitsuhashi 1975), *Lactobacillus casei* (Chassy, Gibson and Guiffrida 1978), *L. reuteri* (Axelsson *et al.*^1988^) and *Oenococcus oeni* (used in wine production) (Mesas, Rodriguez and Alegre 2004) (Table 1). Acriflavine was effective at curing resistance from antibiotic-resistant *Enterococcus faecium* and *E. faecalis* (Coleri *et al.*^2004^).

Acriflavine, ethidium bromide and acridine orange caused loss of a plasmid-encoded staphylococcin production in *Staphylococcus* species (Jetten and Vogels 1973; Ersfeld-Dressen, Sahl and Brandis 1984); however, as strains became resistant to acriflavine they also became resistant to its curing effects (Jetten and Vogels 1973). Acriflavine, acridine orange and ethidium bromide cured resistance to antimicrobials from both donor and transconjugants *B. fragilis* and *B. thetaiotaomicron* (Rotimi, Duerden and Hafiz 1981).

The practical applications of DNA intercalating agents are few, due to their activity as powerful mutagens, associated with significant toxicity and the carcinogenic nature of these molecules. The harm of using such compounds vastly outweighs any potential benefit derived from plasmid curing. In addition, as many intercalating agents are substrates of bacterial efflux pumps, the use of such compounds could select for overexpression of efflux pumps which can lead to MDR (Piddock 2006). However, these compounds can still be useful in a laboratory setting to cure strains of plasmids (Coleri *et al.*^2004^; Mesas, Rodriguez and Alegre 2004; Chin *et al.*^2005^; Raja and Selvam 2009; Zaman, Pasha and Akhter 2010; Adeyemo and Onilude 2015; Pulcrano *et al.*^2016^).

### Plant-derived compounds

Many well-studied plant extracts come from traditional medicine. Plumbagin (5-hydroxy-2-methyl-1,4-naphthoquinone) is a yellow dye derived from the root of the tropical/subtropical *Plumbago* species (Patwardhan *et al.*^2015^). Plumbagin is reported to have anticancer, antifungal and antimicrobial activity (Padhye *et al.*^2012^; Tyagi and Menghani 2014). In *E. coli*, plumbagin effectively eliminated a conjugative, MDR plasmid (Lakhmi, Padma and Polasa 1987) and the RP4 plasmid (Bharathi and Polasa 1991). Plumbagin eliminated plasmids from *E. coli*, by decreasing plasmid copy number and reducing the toxic effect of plasmid loss (Lakshmi and Thomas 1996) (Table 1).

Subinhibitory concentrations of *Plumbago zeylanica* root extract were tested on MDR clinical isolates of *S.* Paratyphi*, S. aureus, E. coli* and *Shigella dysenteriae*, as well as *E. coli* containing pUK651, but the extract only cured 14% of *E. coli* of pUK651 (Beg and Ahmad 2000). Subinhibitory concentrations of *P. auriculata* root extracts cured drug-resistance plasmids from *P. aeruginosa*, *E. coli*, *Proteus vulgaris* and *K. pneumoniae*, which were slightly higher than pure plumbagin (Patwardhan *et al.*^2015^) (Table 1).

8-epidiosbulbin E acetate is isolated from the bulbs of *Dioscorea bulbifera*, a plant known in Ayurvedic alternative medicine (Shriram *et al.*^2008^). 8-epidiosbulbin E acetate belongs to the clerodane class of diterpenes. Its antibacterial and curing activity was evaluated, and it cured reference strains of *E. coli*, *B. subtilis*, *P. aeruginosa*, and clinical isolates of *E. coli*, *E. faecalis* and *S. sonnei* with an average efficiency of 34% (Shriram *et al.*^2008^) (Table 1).

The curing activity of the crude extract of *Alpinia galanga* (L.) Swartz, a medicinal plant indigenous to Southeast Asian countries, was tested (Latha *et al.*^2009^). The bioactive fraction containing 1΄-acetoxychavicol acetate was tested on nine bacterial reference strains carrying antibiotic-resistance plasmids. A subinhibitory concentration of crude extract cured plasmids from *S.* Typhi*, E. coli* and *E. faecalis*. Purified 1΄-acetoxychavicol acetate cured MDR plasmids from *S.* Typhi, *P. aeruginosa*, *E. faecalis*, *E. coli* and *B. cereus* (Latha *et al.*^2009^) (Table 1).

Taken together, plant-derived compounds can be effective at curing plasmids *in vitro*; however, more research is needed to confirm spectrum of activity, identify the active components and to determine any toxicity and *in vivo* efficacy.

### Conjugation inhibiting compounds

#### Unsaturated fatty acids

Work from de la Cruz and colleagues has focused on performing high-throughput screens of compounds to search for inhibitors of conjugation (Fernandez-Lopez *et al.*^2005^; Getino *et al*. 2015, 2016; Ripoll-Rozada *et al.*^2016^). Their high-throughput screening method used a *lux* reporter under the control of the *lac* promoter, on a simple conjugative plasmid derived from R388 in *E. coli* (Fernandez-Lopez *et al.*^2005^). The donor carried the *lacI* repressor; thus, luminescence was only produced after conjugation (Fernandez-Lopez *et al.*^2005^). They tested a library of microbial extracts, and showed that unsaturated fatty acids, including dehydrocrypenynic acid, linoleic acid and oleic acid, inhibited conjugation (Fernandez-Lopez *et al.*^2005^). Recently, Lopatkin *et al.* (2017) used linoleic acid to determine the impact of reduced conjugation on plasmid persistence within a population. Indeed, 3.5 μM linoleic acid was sufficient to destabilise a plasmid with low conjugation efficiency from a population; however, it was ineffective for plasmids with higher conjugation efficiencies or which carried a fitness benefit (Lopatkin *et al.*^2017^).

A study of synthetic fatty acids demonstrated that 2-alynoic fatty acids inhibited conjugation; of these, 2-hexadecynoic acid was the most potent, followed by 2-octadecynoic acid (Getino *et al.*^2015^). At concentrations of 0.4 mM, 2-hexadecynoic acid reduced conjugation frequencies of IncW, IncH and IncF plasmids by 100 times, while concentrations of 1 mM were required to reduce conjugation of IncI, IncL/M and IncX plasmids. Conjugation of IncP and IncN plasmids was not affected by 2-hexadecynoic acid. Using molecules with similar structures, they determined that the carboxylic group, a 16-carbon chain and one unsaturated bond were optimal for conjugation inhibition. They showed that 2-hexadecanoic acid acted on the donor, and inhibited conjugation in *E. coli, S. enterica, P. putida* and *Acinetobacter baumannii* (Getino *et al.*^2015^).

Four unsaturated fatty acids (linoleic, oleic, 2-hexadecynoic and 2-ocatadecynoic acid) inhibited the activity of the plasmid encoded TrwD ATPase (VirB11 homologue) (Ripoll-Rozada *et al.*^2016^). TrwD acts as a traffic ATPase, regulating switching between pilus biogenesis and DNA translocation through the conjugation machinery (Ripoll-Rozada *et al.*^2013^). Fatty acids which did not inhibit conjugation had no impact on TrwD activity (Ripoll-Rozada *et al.*^2016^). The authors suggested that the mechanism for the conjugation inhibiting activity of unsaturated fatty acids was due to their binding to the N-terminal domain and linker region of TrwD, inhibiting the movement of the N-terminal domain over the C-terminal domain, thus preventing ATPase activity of the enzyme (Ripoll-Rozada *et al.*^2016^).

One of the concerns about any clinical use of synthetic fatty acids, such as 2-hexadecanoic acid, is toxicity in people or animals. Recent work focused on finding less-toxic molecules by screening a natural compound library produced by aquatic microbes (Getino *et al.*^2016^). Tanzawaic acid A and B, polyketides produced by *Penicillium* species, were identified as effective conjugation inhibitors of IncW and IncFII plasmids. Tanzawaic acid B (0.4 mM) reduced conjugation by 100-fold for IncW and IncFII, as compared to untreated controls. However, they were only moderately effective on IncFI, IncI, IncL/M, IncX and IncH plasmids, reducing conjugation by between 10% and 50% compared to untreated cells. In addition, they did not inhibit conjugation of IncN and IncP plasmids (Getino *et al.*^2016^). Importantly, oleic acid, linoleic acid and tanzawaic acids A and B were less toxic on bacteria, fungi and tissue culture cells than 2-hexadecynoic and 2-oxydecynoic acid (Getino *et al.*^2016^).

Unsaturated fatty acids have been shown to be effective conjugation inhibitors in many laboratory settings, and on a variety of plasmids. Furthermore, they are associated with reduced toxicity on tissue culture cells. Further studies are needed to determine the *in vivo* safety and efficacy of unsaturated fatty acids, but they are promising candidates for future plasmid curing work.

#### TraE inhibitors

Using a targeted approach, Baron and colleagues have identified small molecules which bind to and inhibit the dimerisation of TraE, an essential component of the type IV secretion system involved in a variety of functions including conjugation of pKM101 (Paschos *et al.*^2011^; Casu *et al*. 2016, 2017). Structural studies of the pKM101 encoded TraE dimerisation (VirB8 homologue) were used as a basis for uncovering small molecules which inhibited dimerisation, four of which (molecules B8I-16, BAR-072, BAR-073 and UM-024) also inhibited transmission of pKM101 (Casu *et al.*^2016^). None of these molecules impacted upon transmission of RP4, highlighting their specificity for pKM101 TraE (Casu *et al.*^2016^). In a follow-up study, Casu *et al.* (2017) screened a fragment library for compounds which bound to TraE. They used this information to design two molecules which bound with high affinity to TraE and were able to reduce transmission of pKM101 (molecules 105055 and 239852) (Casu *et al.*^2017^). Together, this work demonstrates the feasibility and specificity of structure-based design of anti-plasmid compounds.

### Drugs used in human medicine

#### DNA gyrase/topoisomerase inhibitors

DNA gyrase is essential in bacteria as it introduces supercoiling into DNA molecules; it is comprised of two GyrA and two GyrB monomers (Andriole 2005). Multiple antibiotics target DNA gyrase. Aminocoumarin antibiotics, such as novobiocin and coumermycin A, inhibit GyrB (Gellert *et al.*^1976^). These and the related compounds clorobiocin and isobutyryl novenamine were effective at plasmid curing (Hooper *et al.*^1984^). The GyrB inhibiting activities of aminocoumarins are responsible for their plasmid curing properties (Taylor and Levine 1979), and the *E. coli* gyrase B subunit is required for plasmid maintenance, and curing activity of coumermycin A1 (Wolfson *et al.*^1982^). Novobiocin interfered with plasmid maintenance, rather than selecting plasmid-free isolates (Hooper *et al.*^1984^). Furthermore, bacteria with a mutation in *gyrB* conferring resistance to coumermycin required higher levels of the antibiotic to produce the curing effect (Hooper *et al.*^1984^).

Novobiocin was effective at curing plasmids from many Gram-positive bacteria including *L. plantarum*, *Lactobacillus* strains isolated from chickens, *L. acidophilus* isolated from molasses, *E. faecalis*, clinical isolates of enterococci, *B. subtilis* and *S. aureus* (Table 2) (McHugh and Swartz 1977; Ruiz-Barba, Piard and Jiménez-Díaz 1991; Chin *et al.*^2005^; Karthikeyan and Santosh 2010). *Escherichia coli* and other Gram-negative *Enterobacteriaceae* were cured of a variety of plasmids by novobiocin (Michel-briand *et al.*^1986^). Novobiocin eliminated the *Salmonella* virulence plasmid from *S.* Typhimurium, resistance plasmids from *Serratia marcescens* and a cryptic plasmid from *Chlamydia muridarum* (Gulig and Curtiss III 1987; Llanes *et al.*^1990^; O’Connell and Nicks 2006). Coumermycin eliminated some plasmids from *E. coli*, but not RP4 (Danilevskaya and Gragerov 1980; Wolfson *et al.*^1982^; Bharathi and Polasa 1991).

**Table 2.** Quinolone and aminocoumarin antimicrobials with plasmid curing properties.

Quinolone	Species	Plasmid cured	Key findings	Reference
Ciprofloxacin	*E. coli*	R446b	1/2 MIC: no curing, 0.06 μg/mL (sub-MIC): 30% CF	Weisser and Wiedemann (1985); Michel-briand *et al.* (1986)
		R386	0.07 μg/mL (sub-MIC): 2% CF	Michel-briand *et al.* (1986)
		F’lac	1/2 MIC: 50% CF	Weisser and Wiedemann (1985)
		R16	1/2 MIC: 1% CF	Weisser and Wiedemann (1985)
		Rts1	1/2 MIC: 32% CF	Weisser and Wiedemann (1985)
		5 large plasmids	Sub-MIC: 10%–90% CF. Small high copy plasmids not cured	Platt and Black (1987)
	*S. sonnei*	pWR105	0.05 μg/mL (sub-MIC): 50% CF	Michel-briand *et al.* (1986)
Coumermycin A	*E. coli*	pBR322	5 μg/mL: 90% CF, 7 μg/mL: 45% CF. Mechanism involves antagonism of DNA gyrase	Danilevskaya and Gragerov (1980); Wolfson *et al.* (1982)
		pMG110	7 μg/mL: 70% CF and mechanism involves antagonism of DNA gyrase.	Wolfson *et al.* (1982)
		pMB9	5 μg/mL: 64.7% CF. Cou resistant mutant had 5% CF at 10 μg/mL	Danilevskaya and Gragerov (1980)
		pOD162	5 μg/mL: 64.5% CF	Danilevskaya and Gragerov (1980)
		pSC101	2 μg/mL: 32.5% CF	Danilevskaya and Gragerov (1980)
		pKT231	3.15 μg/mL: 90% CF	Bharathi and Polasa (1991)
		pRK2013	3.15 μg/mL: 35.5% CF	Bharathi and Polasa (1991)
Enoxacin	*E. coli*	R446b	1/2 MIC: 24% CF, 0.5 μg/mL (sub-MIC): 2% CF	Weisser and Wiedemann (1985); Michel-briand *et al.* (1986)
		R386	0.05 μg/mL (sub-MIC): 2% CF	Michel-briand *et al.* (1986)
		S-a	0.5 μg/mL (sub-MIC): 1% CF	Michel-briand *et al.* (1986)
		F’lac	1/2 MIC: 66% CF	Weisser and Wiedemann (1985)
		R16	1/2 MIC: 11% CF	Weisser and Wiedemann (1985)
		Rts1	Sub-MIC concentrations: 98% CF	Weisser and Wiedemann (1985)
		pORF2	Sub-MIC concentrations: 43% CF	Fu *et al.* (1988)
	*S. sonnei*	pWR105	0.12 μg/mL (sub-MIC): 11% CF	Michel-briand *et al.* (1986)
Flumequine	*E. coli*	R446b	8 μg/mL (sub-MIC): 2% CF	Michel-briand *et al.* (1986)
		S-a	4 μg/mL (sub-MIC): 1% CF	Michel-briand *et al.* (1986)
	*S. sonnei*	pWR105	0.25 μg/mL (sub-MIC): <1% CF	Michel-briand *et al.* (1986)
	*S. dysenteriae*	pWR24	0.12 μg/mL (sub-MIC): 2% CF	Michel-briand *et al.* (1986)
	*S. flexneri*	PWR110	0.12 μg/mL (sub-MIC): <1% CF	Michel-briand *et al.* (1986)
Nalidixic Acid	*E. coli*	pMG110	4.3 μM (sub-MIC): 1% CF	Hooper *et al.* (1984)
		R446b	1/2 MIC: 8% CF, 64 μg/mL (sub-MIC): 4% CF	Weisser and Wiedemann (1985); Michel-briand *et al.* (1986)
		F’lac	1/2 MIC: 18% CF	Weisser and Wiedemann (1985)
		R16	1/2 MIC: 41% CF	Weisser and Wiedemann (1985)
		Rts1	1/2 MIC: 4% CF	Weisser and Wiedemann (1985)
		pMC1314	Sub-MIC concentrations of 0.3 μg/mL: 9.6% CF; 0.6 μg/mL: 17% CF; 1.2 μg/mL: 36% CF	Courtright, Turowski and Sonstein (1988)
		S-a	32 μg/mL (sub-MIC): 1.5% CF	Michel-briand *et al.* (1986)
	*S. sonnei*	pWR105	8 μg/mL (sub-MIC): 1% CF	Michel-briand *et al.* (1986)
	*S. enterica* Typhimurium	R1 plasmids	6.25 μM eliminated resistance with CFs of: 70% Kan, 56% Chl, 60% Str, 64% Amp	Hahn and Ciak (1976)
Norfloxacin	*E. coli*	R446b	1/2 MIC: 18% CF, 0.1 μg/mL (sub-MIC): 1% CF	Weisser and Wiedemann (1985); Michel-briand *et al.* (1986)
		S-a	0.25 μg/mL (sub-MIC): 3% CF	Michel-briand *et al.* (1986)
		F’lac	1/2 MIC: 19% CF	Weisser and Wiedemann (1985)
		R16	1/2 MIC: 25% CF	Weisser and Wiedemann (1985)
		Rts1	1/4 MIC: 52% CF	Weisser and Wiedemann (1985)
	*S. sonnei*	pWR105	0.5 μg/mL (sub-MIC): <1% CF	Michel-briand *et al.* (1986)
Novobiocin	*E. coli*	pDT4	Novobiocin-sensitive strain was cured, but isogenic resistant strain was not	Taylor and Levine (1979)
		pMG110	22 μM: 99% CF in wild-type strain, in *gyrB* resistant strain 990 μM: 33.3% CF	Hooper *et al.* (1984)
		R386	200 μg/mL: 15% (IncFI) CF	McHugh and Swartz (1977)
		R1–16	175 μg/mL: 34% (IncFII) CF	McHugh and Swartz (1977)
		R726	175 μg/mL: 16.1% (IncH) CF	McHugh and Swartz (1977)
		pMG102	50 μg/mL: 20.3%, 100 μg/mL: 14.7% CF	McHugh and Swartz (1977)
	*S. enterica*	Virulence plasmid (100 kb)	200–250 μg/mL used to cure virulence plasmid	Gulig and Curtiss III (1987)
	*Enterobacter*	pMG150	225 μg/mL: 52.5% CF	McHugh and Swartz (1977)
	*E. faecalis*	pJH1	8 μg/mL: 34% CF	McHugh and Swartz (1977)
	*Enterococcus*	pDR1	10 μg/mL: 28% CF	McHugh and Swartz (1977)
	*L. plantarum*	Multiple unidentified plasmids (2–68 kb)	0.125–0.25 μg/mL: 94%–100% CF for four isolates	Ruiz-Barba, Piard and Jiménez-Díaz (1991)
	*L. fermentum*	Ery resistance plasmid	1.8–40 μg/mL (sub-MIC): 64% CF, and 2.1% CF for two strains	Chin *et al.* (2005)
	*L. acidophilus*	Ery resistance plasmids (4.4–11.5 kb)	1.8–40 μg/mL (sub-MIC): 3.3%–9.0% CF	Chin *et al.* (2005)
		Chl resistance plasmid (20.3 kb)	2.4 μg/mL: 4.6% CF, peaked at 18 h	Karthikeyan and Santosh (2010)
	*C. muridarum*	Cryptic plasmid (7.5 kb)	4%–30% effective, but optimal concentration inhibited 99% of bacterial growth	O’Connell and Nicks (2006)
Ofloxacin	*E. coli*	R446b	1/2 MIC: 10% CF	Weisser and Wiedemann (1985); Michel-briand *et al.* (1986)
		F’lac	1/2 MIC: 39% CF	Weisser and Wiedemann (1985)
		R16	1/2 MIC: 19% CF	Weisser and Wiedemann (1985)
		Rts1	1/4 MIC: 32% CF	Weisser and Wiedemann (1985)
Oxolinic acid	*E. coli*	pMC1314	Sub-MIC concentrations of 0.06 μg/mL: 24% CF; 0.12 μg/mL: 36% CF; 0.25 μg/mL: 100% CF	Courtright, Turowski and Sonstein (1988)
Pefloxacin	*E. coli*	R446b	1/2 MIC: 21% CF, 0.1 μg/mL(sub-MIC): 1% CF	Weisser and Wiedemann (1985); Michel-briand *et al.* (1986)
		F’lac	1/2 MIC: 6% CF	Weisser and Wiedemann (1985); Selan *et al.* (1988)
		R16	1/2 MIC: 16% CF	Weisser and Wiedemann (1985)
		Rts1	1/2 MIC: 27% CF	Weisser and Wiedemann (1985)
	*S. sonnei*	pWR105	1 μg/mL (sub-MIC): 2% CF	Michel-briand *et al.* (1986)
	*S. dysenteriae*	pWR24	1 μg/mL (sub-MIC): 4% CF	Michel-briand *et al.* (1986)
	*S. flexneri*	PWR110	1 μg/mL (sub-MIC): 4% CF	Michel-briand *et al.* (1986)
Pipemidic acid	*E. coli*	R446b	1/2 MIC: 4% CF4 μg/mL (sub-MIC): 6% CF	Weisser and Wiedemann (1985); Michel-briand *et al.* (1986)
		F’lac	1/2 MIC: 35% CF	Weisser and Wiedemann (1985)
		R16	1/2 MIC: 31% CF	Weisser and Wiedemann (1985)
		Rts1	1/2 MIC: 47% CF	Weisser and Wiedemann (1985)
		R386	2 μg/mL (sub-MIC): 0.5% CF	Michel-briand *et al.* (1986)
		S-a	4 μg/mL (sub-MIC): 1% CF	Michel-briand *et al.* (1986)
	*S. sonnei*	pWR105	1 μg/mL (sub-MIC): no curing	Michel-briand *et al.* (1986)
Trovafloxacin	*E. coli*	pT713 (partial)	MIC: 50% CF	Brandi, Falconi and Ripa (2000)
		pJEL144 (partial)	⅓ MIC: 50% CF	Brandi, Falconi and Ripa (2000)
		pRK2 (partial)	1/2 MIC: 30% CF. Also reduced copy number	Brandi, Falconi and Ripa (2000)
Other Quinolones	*E.coli*	R446b	**Rosoxacin**: 2 μg/mL (sub-MIC): 1% CF **β-Hydroxypiromydic acid**: 32 μg/mL (sub-MIC): 3% CF **Cinoxacin**: 4 μg/mL (sub-MIC): 1% CF	Michel-briand *et al.* (1986)
		R386	**Rosoxacin**: 0.05 μg/mL (sub-MIC): 0.5% CF **β-Hydroxypiromydic acid**: 4 μg/ml (sub-MIC): 0.5% CF **Cinoxacin**: 4 μg/mL (sub-MIC): 0.5% CF	Michel-briand *et al.* (1986)
		S-a	**Rosoxacin**: 2 μg/mL (sub-MIC): 1% CF **β-Hydroxypiromydic acid**: 64 μg/mL (sub-MIC): no curing **Cinoxacin**: 4 μg/mL (sub-MIC): 1% CF	Michel-briand *et al.* (1986)
	*S. sonnei*	pWR105	**Rosoxacin**: 0.12 μg/mL (sub-MIC): <1% CF **β-Hydroxypiromydic acid**: 0.25 μg/mL (sub-MIC): <1% CF **Cinoxacin**: 1 μg/mL (sub-MIC): 12% CF	Michel-briand *et al.* (1986)

CF—Curing Frequency: the proportion of colonies which were cured of the plasmid compared to non-cured colonies. Kan—kanamycin, Chl—chloramphenicol, Str—streptomycin, Amp—ampicillin, Cou—coumermycin.

Quinolone antimicrobials also target DNA gyrase. There have been numerous reports of plasmids cured from various bacterial species by different quinolone antibiotics (Table 2). The majority of studies have been done using *E. coli.* For example, five fluoroquinolones and two quinolones cured four plasmids (Weisser and Wiedemann 1985), and subinhibitory levels of quinolones cured *E. coli* of various plasmids including large clinical plasmids (Table 2) (Oliva *et al.*^1985^; Platt and Black 1987; Courtright, Turowski and Sonstein 1988; Selan *et al.*^1988^). However, quinolones have variable curing activity on some plasmids (Weisser and Wiedemann 1985, 1986). For example, quinolones resulted in incomplete curing and reduced copy number of several plasmids (Table 2) (Phillips and Towner 1990; Brandi, Falconi and Ripa 2000), and were ineffective at curing *E. coli* of other plasmids (pBP1, R391, R27 or three small, high-copy plasmids from a clinical *E. coli* isolate) (Weisser and Wiedemann 1985; Platt and Black 1987). In line with this, one study demonstrated in *E. coli* that quinolones cured pORF2 with high efficiency, three plasmids were poorly cured and three plasmids were unaffected (Table 2) (Fu *et al.*^1988^). Interestingly, this study also examined quinolone efficacy at curing pORF2 from *E. coli in vivo.* They found quinolone treatment of mice infected with *E. coli/*pORF2 led to significant reduction in plasmid carriage (Fu *et al.*^1988^).

In a large study, 12 quinolones were tested for their ability to cure 11 plasmids of different incompatibility groups from *E. coli*, and virulence plasmids in five other species of *Enterobacteriaceae* (Table 2) (Michel-briand *et al.*^1986^). The authors concluded that non-fluorinated quinolones had slightly higher curing activity, but that novobiocin cured better than quinolones (Michel-briand *et al.*^1986^). Other studies examining a range of bacteria showed subinhibitory concentrations of quinolones reduced resistance and virulence plasmids in *S. aureus, S.* Typhimurium, *E. coli, P. aeruginosa* and *Yersinia pseudotuberculosis* (Hahn and Ciak 1976; Sonstein and Burnham 1993).

In summary, aminocoumarin-mediated curing appears to be more effective on Gram-positive bacteria than Gram-negative bacteria. Quinolone-mediated plasmid curing is effective on some plasmids in Gram-negative bacteria such as *E. coli.* However, this is complicated by the presence of plasmid-mediated quinolone-resistance genes, such as *qnr, aac*(6΄)-*1b-cr, qepA* and *oqxAB* (Jacoby, Strahilevitz and Hooper 2014; Rodriguez-Martinez *et al.*^2016^). Attempting to use quinolones to cure plasmids carrying quinolone-resistance genes could provide a fitness advantage to plasmid-containing cells, and would therefore select for plasmid maintenance. Furthermore, plasmid-mediated quinolone-resistance genes are frequently coded for by plasmids which carry other resistance genes conferring resistance to antimicrobials including beta-lactams, extended spectrum beta-lactams, carbapenems, aminoglycosides, trimethoprim and chloramphenicol (Rodriguez-Martinez *et al.*^2016^). Taken together, it is unlikely that antibiotics will be used to cure AMR plasmids in humans, animals or the environment as this will provide selection pressure for resistance to arise or be maintained within bacteria. Therefore, aminocoumarin and quinolone antibiotics are an effective laboratory tool, but are unlikely to be used elsewhere for plasmid curing.

#### Rifampicin

The antibiotic rifampicin inhibits RNA polymerase and is used to treat tuberculosis. Subinhibitory concentrations of rifampicin cured a penicillin-resistance plasmid from *S. aureus* (Johnston and Richmond 1970) and the F’lac plasmid from *E. coli* (Bazzicalupo and Tocchini-Valentini 1972). However, a rifampicin-resistant strain was not susceptible to curing, suggesting that the mechanism of curing was dependent upon the interaction of rifampicin with RNA polymerase (Bazzicalupo and Tocchini-Valentini 1972). In *E. coli*, haemolysin production was effectively cured by rifampicin (Mitchell and Kenworthy 1977). A gentamicin-resistance plasmid was not cured from two *S. aureus* strains using rifampicin, but it did cure one strain of a penicillin-resistance plasmid (Wood, Carter and Best 1977). Multiple studies have found rifampicin to be less effective than other curing agents (Rubin and Rosenblum 1971; Poppe and Gyles 1988), and given the importance of rifampicin in treating infections such as tuberculosis, it is unlikely to be used as a general plasmid curing agent.

#### Ascorbic acid

Research on the bioactive compound ascorbic acid (vitamin C) dates back to the first half of the 20th century. In aerobic conditions, ascorbic acid converts circular covalently closed DNA into open circular DNA (Morgan, Cone and Elgert 1975). To investigate the mechanism of action, fragments of pBR322 with radio-labelled 3΄ ends were used to demonstrate that efficient cleavage occurred preferentially at purine-rich regions (Chiou *et al.*^1985^). Studies on DNA extracted from *E. coli* demonstrated that ascorbic acid specificity was linked to negative torsion of the DNA, and this was influenced by ionic strength, salt concentration and pH (Wang and Ness 1989). *In vitro* studies on plasmid pBR322 DNA showed that ascorbic acid increased the damaging effects of dimethylarsinous acid and human liver ferritin (Ahmad, Kitchin and Cullen 2002). Synthesised ascorbic acid variants with protected (non-reactive) hydroxyl groups were tested for their ability to relax pUC19, which demonstrated that the hydroxyl groups at position C2 and C3 were essential for DNA damage (Liu *et al.*^2006^).

In *S. aureus* 1 mM ascorbic acid resulted in loss of penicillin and aminoglycoside resistance encoding plasmids (Table 1) (Amábile Cuevas 1988; Amábile-Cuevas, Piña-Zentella and Wah-Laborde 1991). Two plasmids, pI258 (penicillin resistance) and pT181 (tetracycline resistance), were not cured by ascorbic acid. However, there was a significant decrease in the MIC of tetracycline, which the authors hypothesised was due to reduction in plasmid copy number (Amábile-Cuevas, Piña-Zentella and Wah-Laborde 1991).

Ascorbic acid (1 mM) cured the lactic acid bacterium *Pediococcus acidilactici* of a plasmid coding for the production of pediocin, a metabolite which inhibits growth of some pathogenic bacteria, thus minimising food spoilage (Ramesh, Halami and Chandrashekar 2000). Ascorbic acid is non-toxic and is associated with human health benefits. This makes it an attractive curing agent, although it seems to be more effective at curing plasmids from Gram-positive rather than Gram-negative bacteria. Furthermore, after vitamin C supplementation concentrations of ascorbic acid in the plasma are relatively low (0.07 mM), but concentrations in lymphocytes can be much higher (3.5 mM), and concentrations in duodenal biopsies were around 1.2 mmol/kg (Levine *et al.*^1996^; Waring *et al.*^1996^). Conversely, Maier *et al.* (2018) estimated ascorbic acid concentrations in the intestine to be around 0.379 mM. Together this shows that while plasma concentrations after supplementation would not reach sufficient levels to have anti-plasmid activity, ascorbic acid is concentrated in the intestine, where it could potentially affect plasmids within intestinal bacteria. However this remains to be demonstrated *in vivo.*

#### Psychotropic drugs

The phenothiazines have been widely used in human medicine, originally as anti-helminthics, but now this class of drugs comprises the largest of five classes of anti-psychotic drugs (Ohlow and Moosmann 2011). The impact of these molecules on bacteria has been reviewed elsewhere (Amaral, Viveiros and Molnar 2004; Spengler *et al.*^2006^; Varga *et al.*^2017^). Plasmid curing properties have also been attributed to phenothiazines (Table 1) (Amaral, Viveiros and Molnar 2004; Spengler *et al.*^2006^; Dastidar *et al.*^2013^). In addition, a recent study found that chlorpromazine significantly impacted the growth of diverse members of the human microbiome, including *Akkermansia muciniphila, Bacteroides uniformis, B. vulgatus, Clostridium perfringens*, *Parabacteroides distasonis* and *P. merdae* (Maier *et al.*^2018^). Phenothiazines, including chlorpromazine, cured plasmids from *E. coli* (Table 1) (Mandi *et al.*^1975^; Molnar, Mandi and Kiraly 1976), and the curing activity was enhanced by methylene blue (Molnar *et al.*^1980^). Thioridazine cured the AMR phenotype from *E. coli*, *S. flexneri* and *V. cholerae* isolates, but not from *S. aureus* (Table 1) (Radhakrishnan *et al.*^1999^), while promethazine and trifluoperazine were tested on clinical isolates of *E. coli, Citrobacter freundii* and *E. cloacae*, but only one *E. coli* isolate was cured, despite *E. coli* K12 being readily cured of a lac-reporter plasmid (Spengler *et al.*^2003^). However, trifluoroketone 18 or trifluoromethyl-ketone 14 (proton pump inhibitors) enhanced curing activity of the phenothiazines, suggesting the compounds may be effluxed (Spengler *et al.*^2003^; Wolfart *et al.*^2006^). In mixed cultures of *E. coli, B. cereus* and *S. epidermidis*, promethazine cured F’lac from *E. coli* (Molnár, Amaral and Molnár 2003). Chlorpromazine cured the MRSA Iberian clone strain HPV107 of a plasmid encoding the QacA efflux pump (Costa *et al.*^2010^).

Together, this shows phenothiazines have *in vitro* curing activity on some bacteria and plasmid combinations. However, their *in vivo* efficacy as plasmid curing compounds remains unclear. Any potential connection between anti-plasmid and the anti-commensal activity of chlorpromazine remains to be elucidated. In patients being treated for psychosis with chlorpromazine, serum concentrations are around 0.1–0.3 μg/mL, and toxic side effects occur at 0.75 μg/mL (Sanofi-Aventis 2016). However, Maier *et al.* (2018) estimate intestinal concentration of chlorpromazine to be around 46 μM (14.67 μg/mL). The concentrations used for plasmid curing are generally around 10–100 μg/mL (Mandi *et al.*^1975^; Spengler *et al.*^2003^). Therefore, concentrations resulting in curing may be reached in the intestines of individuals being treated with chlorpromazine. Novel approaches involving targeted drug delivery or preventing uptake of orally administered phenothiazines may help to improve curing efficacy and reduce toxicity. Until such obstacles are overcome, the use of phenothiazines for *in vivo* plasmid curing is unlikely.

## INCOMPATIBILITY-BASED PLASMID CURING SYSTEMS

Curing based upon the principle of plasmid incompatibility is an alternative method to chemical or drug-based strategies to remove plasmids from bacteria. Plasmid curing using an incompatible plasmid vector has been widely used in plasmid characterisation of Gram-positive and Gram-negative species. Introducing a smaller high-copy-number plasmid from the same incompatibility group may specifically eliminate a resident plasmid (Bringel, Frey and Hubert 1989). Incompatibility-based curing has been useful for investigating incompatibility mechanisms, plasmid–host interactions and for the construction of gene transfer systems (Uraji, Suzuki and Yoshida 2002). The main advantage of this method is the reduced risk of chromosomal mutations and toxicity sometimes associated with chemical curing agents (Hovi *et al.*^1988^; Poppe and Gyles 1988). In addition, incompatibility-based curing is specific to plasmids of the targeted incompatibility group. One major drawback of incompatibility-based curing methods is the extensive cloning required for set up, and the detailed knowledge of the target plasmid. Ni *et al.* (2008) reported the main difficulty in constructing incompatibility plasmids for curing is the replication control and/or partition region of the plasmid must be identified before curing (Ni *et al.*^2008^). Additional plasmid genes (e.g. antitoxin from a TA system) may need to be included (Ni *et al.*^2008^; Hale *et al.*^2010^).

Incompatibility-based curing has been used in a variety of bacteria and plasmids (Table 3). In particular, when chemical curing methods have proven less effective, e.g. *Lactobacillus*, and *Y. pestis* (Ruiz-Barba, Piard and Jiménez-Díaz 1991; Chin *et al.*^2005^; Ni *et al.*^2008^; Karthikeyan and Santosh 2010). Incompatibility-based curing systems were designed and used in *L. acidophilus, L. plantarum* and *L. pentosus* (Table 3) (Bringel, Frey and Hubert 1989; Posno *et al.*^1991^). Incompatibility has been used to study the contribution of plasmids to bacterial pathogenesis, including a systematic investigation of the role of plasmids in *Y. pestis* pathogenesis (Table 3) (Ni *et al.*^2008^). Incompatibility was used to cure vaccine and wild-type strains of *B. anthracis* of two large pathogenicity-related plasmids (Table 3), allowing study of their contribution to capsule and anthrax toxin production (Wang *et al.*^2011^; Liu *et al.*^2012^). Incompatibility has been used not only in human pathogens, but also to remove tumour inducing (Ti) plasmids from *Agrobacterium tumefaciens*, a dicotyledonous plant pathogen, in which Ti plasmids are responsible for inducing vegetable tumours (Table 3) (Uraji, Suzuki and Yoshida 2002).

**Table 3.** Incompatibility based curing plasmids.

Species	Curing plasmid details	Cured Plasmid Details	Key Findings	Delivery	Reference
*L. plantarum*	**pULP8** and **pULP9** 6.6 kb, Amp and Ery resistance. Constructed by inserting the Ery-resistance gene from pVA891 into a pUC19-pLP1 construct	2.1 kb pLp1 endogenous plasmid	Maintained in 5% of bacteria after 20 generations (selection free media). TE: 2 × 10^−7^ CFU/μg DNA	Electroporation	Bringel, Frey and Hubert (1989)
*L. pentosus*	**pLP3537**, 6.3 kb, Ery resistance. Constructed by inserting 2.3 kb endogenous plasmid into a screening vector, pEI2. Contained lactobacillus replicon	2.3 kb endogenous plasmid	Maintained in 8% of bacteria after 100 generations (selection free media). TE: 10^2^–10^3^ CFU/μg DNA	Electroporation	Posno *et al.* (1991)
	**pLPE323**, 3.6 kb, Ery resistance. Constructed by inserting 2.3 kb endogenous plasmid into pE194 vector. Contained lactobacillus replicon	2.3 kb endogenous plasmid	Maintained in 100% of bacteria after 100 generations (selection free media). TE: 10^2^–10^3^ CFU/μg DNA	Electroporation	Posno *et al.* (1991)
	**pGK12**, 4.4 kb, broad Gram-positive host range plasmid	1.7 kb endogenous plasmid	Maintained in <1% of bacteria after 100 generations (selection-free media). TE: 10^3^	Electroporation	Posno *et al.* (1991)
*A. tumefaciens*	**pMGTrep1**, contained pTi *repABC* genes and *sacB* (sucrose sensitivity gene) to select for pMGTrep1 loss	pTi-SAKURA (206kb) pTiC58 (214kb)	Between 32% (pTi-SAKURA) and 99% (pTiC58) of transconjugants were cured of pTi	Conjugation	Uraji, Suzuki and Yoshida (2002)
*Y. pestis*	**pEX18-PCP-** pPCP1 replicon, *sacB*	pPCP1 virulence plasmid (ColE1)	64% of colonies cured	Electroporation	Ni *et al.* (2008)
	**pEX18-MT-** pMT1 replicon, *sacB*	pMT1 virulence plasmid (*repA*)	30% of colonies cured	Electroporation	Ni *et al.* (2008)
	**pEX18-CD-** pCD1 replicon, *sacB*	pCD1 virulence plasmid (IncFIIA)	98% of colonies cured	Electroporation	Ni *et al.* (2008)
	**pEX18-CRY-** pCRY replicon, *sacB*	pCRY (21.7 kb) cryptic plasmid	70% of colonies cured	Electroporation	Ni *et al.* (2008)
*B. anthracis*	**pKS5K**, contains ORF43–46, temperature sensitive	pXO1 (181.6 kb) encodes anthrax toxin/regulatory genes (*pagA, lef, cya,atxA, pagR*)	Isolate was successfully cured. CF not determined	Electroporation	Liu *et al.* (2012)
	**pKSV7-*oriIV***, contains pXO2 *repS, repB, ori* sequences, temperature sensitive	pXO2 (93.5 kb) encodes capsule synthesis and degradation genes (*capABCD*).	Isolate was successfully cured. CF not determined	Electroporation	Wang *et al.* (2011)
	**pKORT**, derived from pKSV7, contains pXO1 and pXO2 origins of replication, temperature sensitive	pXO1 and pXO2	Isolate was successfully cured. CF not determined	Electroporation	Wang *et al.* (2015)
*E. coli*	**pCURE1**, anti-pO157, pMB1 replicon, *oriT*_RK2_, *sacB*, Amp and Kan resistance	pO157 (F-like plasmid)	Isolate was successfully cured. CF not determined	Transformation or mobilisation by IncP-1 transfer system (due to *oriT*_RK2_)	Hale *et al.* (2010)
	**pCURE2**, anti-IncF pMB1 replicon, *oriT*_RK2_, *sacB*, Amp and Kan resistance	IncF-like plasmids including p1658/97, pKDSC50 (RepFIB and RepFIIA), F and F’ plasmids (RepFIA)	Highly effective on IncF plasmids, CF up to 100%. Inclusion of anti-toxin genes on pCURE2 increased efficacy	Transformation or mobilisation by IncP-1 transfer system (due to *oriT*_RK2_)	Hale *et al.* (2010)
	**pCURE11**, anti-IncP-1α, pMB1 replicon, *oriT*_RK2_, *sacB*, Amp and Kan resistance	pRK24 (IncP-1α), derivative of RK2	CF: 100% of tested colonies	Transformation or mobilisation by IncP-1 transfer system (due to *oriT*_RK2_)	Hale *et al.* (2010)
	**pJIMK3**, *pemI* anti-toxin gene, no incompatibility genes included	pEI1573 (IncL/M), carries *bla*_IMP-4_ isolated from *E. cloacae*	30% CF of pEI1573 in *E. coli*	Transformation	Kamruzzaman *et al.* (2017)
	**pJIMK25**, pJIMK3 with addition of IncL/M replication fragments	pEI1573	100% CF pEI1573 in *E. coli*	Transformation	Kamruzzaman *et al.* (2017)
*E. coli, K. pneumoniae, C. freundii, M. morganii*	**pJIMK46**, *pemI* anti-toxin gene, *fosA3*, IncL/M and IncI1 replicons	pEI1573, pJIE512b (conjugative IncI1 plasmid with *bla*_CMY-2_ isolated from *E. coli*)	Cured when curing plasmid was selected for using antibiotics. Cured *in vitro* in *E. coli, K. pneumoniae, C. freundii, M. morganii.* Cured pEI1573 *in vivo*, from *E. coli*, but required antibiotic selection for pJIMK46	Conjugation	Kamruzzaman *et al.* (2017)

TE—Transformation efficiency of curing plasmid, CF—curing frequency of plasmid, Ery—erythromycin, Amp—ampicillin, Kan—kanamycin.

Incompatibility-based plasmids called pCURE were constructed for curing pO157 (a typical F-like plasmid), other F-like and IncP-1α plasmids from *E. coli* (Table 3) (Hale *et al.*^2010^). To create the pCURE constructs, elements expected to interfere with specific functions were chosen, such as repressing vital components (e.g. transcriptional repressor, antisense RNA or other translational regulators) and competition for vital steps (e.g. replication origin) (Hale *et al.*^2010^). To control the TA system, either the putative antitoxin or antisense RNA repressor was included (Hale *et al.*^2010^).

In a recent study, ‘interference plasmids’ were designed which combined an antitoxin gene and replicon genes to cure *bla*_IMP-4_ and *bla*_CMY-2_ encoding plasmids both *in vitro* and *in vivo* (Table 3) (Kamruzzaman *et al.*^2017^). In the presence of the antibiotic selecting for the interference plasmid, target plasmids were effectively removed from *E. coli, K. pneumoniae, C. freundii* and *Morganella morganii in vitro*, and from *E. coli* colonising the mouse intestine. Interference plasmids were lost from the mouse intestine after cessation of antibiotic treatment.

One targeted approach sought small molecules which mimic the incompatibility system of IncB plasmids (Denap *et al.*^2004^). They found the aminoglycoside apramycin binds to the SLI region of the RepA mRNA, preventing translation of RepA, which is necessary for plasmid replication (Denap *et al.*^2004^). Treatment of *E. coli* harbouring pMU2403 (IncB) with apramycin resulted in almost complete plasmid elimination (Denap *et al.*^2004^).

An important question regarding use outside the laboratory of incompatibility-based curing systems is how to apply the curing plasmids to people, animals or the environment. Plasmids could be delivered via bacteria or phage. However, the potential requirement for antibiotic treatment to select for the curing plasmids (Kamruzzaman *et al.*^2017^) would be a significant drawback. Another concern regarding curing plasmids is the potential for acquisition of ARG(s) onto the curing backbone. More research is needed in increasingly complex plasmid systems to study the dynamics between curing plasmids and AMR plasmids, including research focused on minimising the need for antibiotic selection.

## PHAGE-BASED ANTI-PLASMID SYSTEMS

For the past 50 years, bacteriophages which specifically target the pili of plasmid conjugation systems have been studied (Caro and Schnös 1966). More recently, this has been studied in the context of AMR plasmids. Phages which target the conjugation pilus preferentially kill bacteria with high pilus expression (Dionisio 2005). Low pilus expression results in reduced susceptibility to phage, but also reduced conjugation rates. Therefore, diversity in pilus expression within a bacterial population improves the chances of plasmid survival (Dionisio 2005). Another example of bacteriophages specifically targeting AMR plasmids involved the phage PRD1 which targeted the mating pair complex of plasmids RP4 and RN3 (Jalasvuori *et al.*^2011^). PRD1 reduced plasmid carriage within *E. coli* and *Salmonella* populations from 100% to 5% after 10 days. Furthermore, the 5% which retained plasmid had lost the ability to conjugate (Jalasvuori *et al.*^2011^). PRD1 significantly reduced the number of *E. coli* K12 RP4 transconjugants, and even reduced transconjugants when single, sub-MIC antibiotic selection was applied (Ojala, Laitalainen and Jalasvuori 2013). However, when double selection for transconjugants was applied phage-resistant mutants arose, but 65% had lost the ability to conjugate (Ojala, Laitalainen and Jalasvuori 2013). Together, this demonstrates the use of phage to produce an evolutionary pressure which results in either plasmid loss or evolution of a non-conjugative plasmid. This fits with the other research focused on using phage-mediated directed evolution to select for antibiotic sensitive bacteria (Chan *et al.*^2016^).

The M13 filamentous phage minor coat protein g3p was necessary and sufficient to inhibit F-plasmid conjugation in *E. coli* (Lin *et al.*^2011^). Another study modelled the dynamics of the F-plasmid and M13 phage in *E. coli* (Wan and Goddard 2012). They found M13 infection reduced cell growth rate, and the conjugation rate was only one order of magnitude faster than the rate of phage infection. This implies that a high concentration of phage would be required to effectively prevent conjugation, and they showed that conjugation continues even with phage (Wan and Goddard 2012). Recently, the evolutionary and ecological implications of lytic bacteriophage predation on plasmid maintenance in a population of *P. fluorescens* were examined (Harrison *et al.*^2015^). They concluded that phage accelerates plasmid loss in the absence of selective pressure (Harrison *et al.*^2015^).

In summary, these studies show that phage can be a highly effective tool for reducing plasmid prevalence within a population. Another advantage of bacteriophage approaches is their status as ‘generally regarded as safe’, which streamlines downstream applications such as use of phage to decolonise surfaces, as a probiotic or use on farms. However, unclear regulatory pathways for use of phage as medication still pose a problem. Another problem associated with phage therapy is bacterial evolution of resistance to phage. By understanding the evolutionary pressures applied to bacteria by phage predation, this evolution can be harnessed to increase susceptibility to antibiotics (Jalasvuori *et al.*^2011^; Ojala, Laitalainen and Jalasvuori 2013; Chan *et al.*^2016^). Future research is needed to further our understanding of the phage-plasmid-host dynamics, to improve upon evolution-optimised approaches and to test these approaches in increasingly complex models.

## CRISPR/CAS-BASED PLASMID CURING SYSTEMS

CRISRP/Cas is a bacterial ‘adaptive immune system’ which allows recognition, degradation and memory of foreign DNA sequences. CRISPR/Cas works as a result of spacer DNA segments coded by the bacteria that are transcribed into crRNA. The crRNA is bound by the Cas protein complex which cleaves nucleic acid sequences matching the crRNA, resulting in double-stranded breaks (Sternberg and Doudna 2015). DNA repair mechanisms can be used to insert a desired sequence into the break (Sternberg and Doudna 2015). In bacteria, double-stranded breaks are often fatal, but combination with traditional recombineering systems such as λ-red can allow for effective genome editing (Peters *et al.*^2015^). The highly specific CRISPR/Cas system has been extensively described and reviewed elsewhere (Jiang and Doudna 2015; Sternberg and Doudna 2015; Wright, Nuñez and Doudna 2016). In a seminal paper, Garneau *et al.* (2010) showed that *Streptococcus thermophiles* isolates which had lost the plasmid pNT1 had acquired new spacer sequences which targeted pNT1. This work demonstrated that CRISPR/Cas acted to remove plasmid DNA from bacteria.

**Figure 2.**
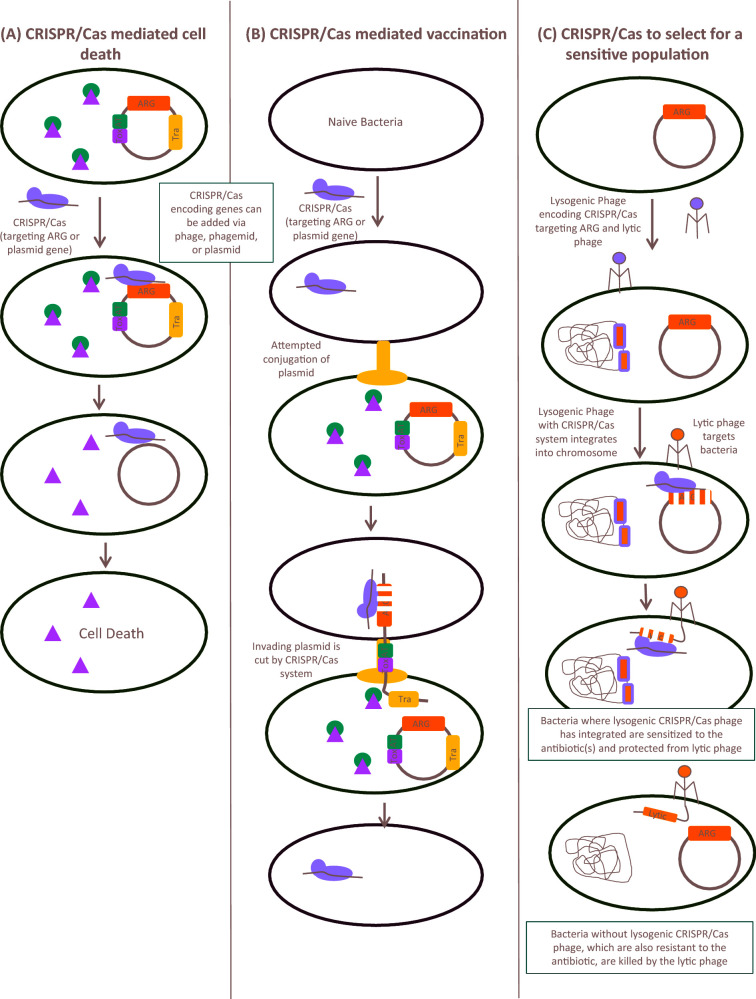
CRISPR/Cas as an anti-plasmid strategy. (**A**) CRISPR/Cas systems (purple) which target plasmid encoded genes cause double-stranded breaks in the AMR plasmid, leading to plasmid degradation. In plasmids with toxin (Tox, blue) antitoxin (AT, green) systems, loss of plasmid leads to active toxin. The toxin then mediates cell death, resulting in removal of AMR plasmid carrying bacteria from a population. (**B**) CRISPR/Cas system prevents uptake of plasmid DNA. Bacteria encoding CRISPR/Cas system that targets plasmid genes degrade incoming DNA, including conjugative (Tra, orange) AMR plasmids, thus preventing spread of AMR palsmids. (**C**) CRISPR/Cas system combined with lysogenic and lytic phages selects for an antimicrobial sensitive population. Lysogenic phages encoding CRISPR/Cas systems which target both AMR plasmid and lytic phage are administered to bacteria. Production of the CRISPR/Cas system results in degradation of the AMR plasmid, and protection from lytic phages. Administration of lytic phages kills all non-sensitised bacteria, which do not encode the lytic phage resistance, thus producing evolutionary pressure for an antimicrobial sensitive population.

Recently, the CRISPR/Cas system has been explored as a method for plasmid curing. Firstly, it can be designed to target specific plasmid genes, including ARGs. The double-stranded breaks introduced in the process can reduce the stability of the plasmid, and in some cases result in plasmid loss (Fig. 2a) (Kim *et al.*^2016^; Lin *et al.*^2016^). Plasmids in isolates from man, animals or the environment frequently carry TA systems. TA systems, sometimes called addiction systems, are comprised of a toxin and an antitoxin gene (Van Melderen and Saavedra De Bast 2009; Chan, Espinosa and Yeo 2016). Generally, the activity of the stable toxin is mitigated by a less stable antitoxin. Therefore, as long as the antitoxin is produced, the toxin cannot act (Van Melderen and Saavedra De Bast 2009; Chan, Espinosa and Yeo 2016). When encoded on plasmids, the TA system functions by killing daughter cells which do not contain a copy of the plasmid coding for the antitoxin gene, a process termed postsegregational killing (Chan, Espinosa and Yeo 2016). Therefore, targeting plasmids with TA systems resulted in bacterial cell death (Fig. 2a) (Citorik, Mimee and Lu 2014). Toxin-mediated cell death could be complemented by antitoxin-encoding phage (Citorik, Mimee and Lu 2014). Specific ARGs can also be targeted by CRISPR/Cas systems (Citorik, Mimee and Lu 2014; Kim *et al.*^2016^). For example, homologous regions in TEM and SHV beta-lactamases were targeted (Kim *et al.*^2016^). CRISPR/Cas systems can also target plasmid backbone genes such as replicase genes (Cao *et al.*^2017^). CRISPR/Cas systems targeted and removed multiple AMR plasmids simultaneously (Yosef *et al.*^2015^).

Secondly, CRISPR/Cas is an attractive strategy because it can be used to prevent plasmid transmission by ‘vaccination’ (Fig. 2b). Methicillin-sensitive *S. aureus* was vaccinated against pUSA02, the plasmid responsible for methicillin resistance in the epidemic MRSA strain USA300 (Bikard *et al.*^2014^). Likewise, *E. coli* containing CRISPR/Cas targeting *bla*_CTX-M-15_ and *bla*_NDM-1_ were less efficiently transformed with an AMR plasmid carrying these genes (Yosef *et al.*^2015^). In *E. faecalis* the CRISPR3-Cas locus was deleted, resulting in significantly higher acquisition of the pAD1 plasmid (target sequence located in CRISPR3), while acquisition of pCF10 was unaffected, as it is not targeted by CRISPR3 (Price *et al.*^2016^). In line with this, carbapenem-resistant *K. pneumoniae* were less likely to have active CRISPR/Cas systems than carbapenem-sensitive strains (Lin *et al.*^2016^). Similarly, type I-F CRISPR/Cas systems were more common in *E. coli* isolates that were antimicrobial sensitive (Aydin *et al.*^2017^). Some of these CRISPR spacers aligned to sequences commonly found in IncFII and IncI1 plasmids, which are associated with clinical resistance (Aydin *et al.*^2017^). This strongly suggests that antimicrobial-sensitive isolates can use CRISPR/Cas systems to degrade incoming AMR plasmids.

The benefits of using CRISPR/Cas systems to cure bacterial plasmids are clear. However, there are significant drawbacks associated with this strategy. One of the primary problems is delivery. A variety of delivery methods including plasmid transformation, conjugation, phagemid and bacteriophages have been used predominantly *in vitro*, with limited studies using *in vivo* models (Bikard *et al.*^2014^; Yosef *et al.*^2015^; Kim *et al.*^2016^). Despite this, so far the practical use of these systems is limited. One study used bacteria sensitised to antibiotics by a lysogenic CRISPR phage which also carried resistance to a lytic phage, thus combining use of the lytic and lysogenic phages to put evolutionary pressure on bacteria to become drug sensitive (Fig. 2c) (Yosef *et al.*^2015^). A recently devised system termed GOTraP enhances DNA transduction to a variety of bacterial species, including *E. coli, S. sonnei* and *K. pneumoniae* (Yosef *et al.*^2017^). Such a strategy could be employed to improve the delivery of anti-ARG or anti-plasmid CRISPR/Cas systems. In another study, a phagemid was effective for treating *S. aureus* skin lesions in mice (Bikard *et al.*^2014^). CRISPR/Cas may be an effective treatment for curing plasmids from surface wounds and burns, but advances in systemic treatment are still required.

The specificity of CRISPR/Cas is both a benefit and a drawback. For example, the lack of common sequences among variants of *bla*_OXA_ and *bla*_CTX-M_ beta-lactamases restricted CRISPR design (Kim *et al.*^2016^). Frequently, plasmid-mediated antibiotic resistance genes have multiple sequence variants, so if CRISPR/Cas sequences were used to kill bacteria there could be selective pressure for mutations in the regions targeted by CRISPR to give a CRISPR-resistant plasmid (Gomaa *et al.*^2014^). Similarly, in a phagemid system, resistance occurred due to deletions of the *cas9* gene on the phagemid (Bikard *et al.*^2014^). Future research must consider the evolutionary pressure, and as some have done (Yosef *et al.*^2015^), design strategies to reduce development of resistance.

## FUTURE OF PLASMID CURING AND ANTI-PLASMID APPROACHES

We and others anticipate that future research will continue in this area, driven in large part by the need to prevent and treat resistant infections (Getino and de la Cruz 2018). Methods to effectively and safely cure plasmids have the potential to diminish the severity of the impact of drug-resistant infections. Currently, few studies have examined curing methods *in vivo*. These studies along with future *in vivo* plasmid curing studies will be crucial in developing methods to sensitise bacteria to existing antibiotics. In the future, it may be that doctors prescribe a plasmid curing agent to help ensure that the antibiotics taken by the patient are effective. Alternatively, a plasmid curing agent could be taken by an individual (e.g. on return from an area where plasmid-mediated drug-resistance is common) as a way of restoring drug-susceptible bacteria to the gastrointestinal microbiome.

The use of plasmid curing strategies in settings other than in humans and animals should not be under appreciated. Reducing the global burden of AMR will require a multifaceted One-Health approach, and curing AMR plasmids from ARG hot spots such as waste water, manure and downstream of pharmaceutical (antibiotic) factories is a viable strategy. Some of the approaches described may be more suited to an environmental or agricultural setting and not for human use. For instance, another potential use of curing strategies could be on farms where livestock are often exposed to antibiotics, and harbour multiple MDR plasmids. Soil, waste water treatment and aquaculture could all be treated with plasmid curing agents to reduce drug resistance.

One concern surrounding plasmid curing is the potential for the development of resistance to anti-plasmid approaches, and the impact of these approaches on the bacterial community structure. Bacteria are constantly evolving, which makes developing ‘evolution-proof’ drugs extremely challenging, but by striving for this gold standard it may be possible to delay resistance (Bell and MacLean 2018). While these ideas apply to antimicrobials, similar principles can applied to plasmid curing strategies. If an anti-plasmid approach kills or produces a fitness defect in the bacteria, it seems likely that resistance will occur. For example, some of the CRISPR/Cas approaches selectively kill resistant bacteria by targeting specific sequences. Mutations in these sequences would then result in survival of the mutants, and expansion due to the resistance phenotypes. Thus, methods to provide selective advantages for the sensitive strains or to guide evolution towards antimicrobial susceptible strains can be utilised to help overcome these challenges. Antibiotics which have curing properties (e.g. quinolones and rifampicin) are obvious examples where resistance would be selected, and therefore should not be used as curing compounds outside the laboratory. Another concern is cell permeability. If plasmids provide a beneficial trait, mutations which reduce permeability or increase efflux may be selected by plasmid curing compounds. Indeed bacteria may increase expression of efflux to remove potentially detrimental plasmid curing compounds from the cells. Such mutations could also produce reduced susceptibility to clinically important antimicrobials. Research should consider and address these concerns.

Altogether, plasmid curing has come a long way, from the use of toxic compounds to novel designer curing methods based on incompatibility or CRISPR/Cas. Further research is now needed to uncover safe and effective means to cure plasmids, particularly in the face of the global AMR crisis.
